# Psychometric Properties of the Nurses Work Functioning Questionnaire (NWFQ)

**DOI:** 10.1371/journal.pone.0026565

**Published:** 2011-11-08

**Authors:** Fania R. Gärtner, Karen Nieuwenhuijsen, Frank J. H. van Dijk, Judith K. Sluiter

**Affiliations:** Coronel Institute of Occupational Health, Academic Medical Center, University of Amsterdam, Amsterdam, The Netherlands; Finnish Institute of Occupational Health, Finland

## Abstract

**Objectives:**

The Nurses Work Functioning Questionnaire (NWFQ) is a 50-item self-report questionnaire specifically developed for nurses and allied health professionals. Its seven subscales measure impairments in the work functioning due to common mental disorders. Aim of this study is to evaluate the psychometric properties of the NWFQ, by assessing reproducibility and construct validity.

**Methods:**

The questionnaire was administered to 314 nurses and allied health professionals with a re-test in 112 subjects. Reproducibility was assessed by the intraclass correlations coefficients (ICC) and the standard error of measurement (SEM). For construct validity, correlations were calculated with a general work functioning scale, the Endicott Work Productivity Scale (EWPS) (convergent validity) and with a physical functioning scale (divergent validity). For discriminative validity, a Mann Whitney U test was performed testing for significant differences between subjects with mental health complaints and without.

**Results:**

All subscales showed good reliability (ICC: 0.72–0.86), except for one (ICC = 0.16). Convergent validity was good in six subscales, correlations ranged from 0.38–0.62. However, in one subscale the correlation with the EWPS was too low (0.22). Divergent validity was good in all subscales based on correlations ranged from (−0.06)–(−0.23). Discriminative validity was good in all subscales, based on significant differences between subjects with and without mental health complaints (p<0.001–p = 0.003).

**Conclusion:**

The NWFQ demonstrates good psychometric properties, for six of the seven subscales. Subscale “impaired decision making” needs improvement before further use.

## Introduction

Work is one of the most important foundations on which a person's life and quality of life is built, next to health, family and social environment [1]. Not only is work an important source of financial income, but it is also important for identity and self-actualization. Thus, functioning well at work is a necessity for well-being. One known factor that threatens good work functioning is health problems [2]. In particular, mental health problems can negatively impact work functioning and are known to be highly prevalent in the working population [3]–[6]. In the working population, the prevalence of psychological distress is 23% [7]. In some occupations, the presence of impaired work functioning demands special attention, such as in the health care sector. First, common mental disorders (CMDs) are more prevalent in this sector than in other (service) sectors [8]. Second, impairments in work functioning in this sector can be serious and are not limited to the employee and the organization, and they present severe risks for patients as well [9], [10].

Since the last decade, occupational health psychology and occupational medicine have focused more and more on impairments in work functioning due to health problems, which is also referred to as presenteeism [2]–[4], [11]–[18]. To gain additional insights into this concept, its causes and its effects, a number of measurement instruments have been developed [6], [19]. In these instruments, impaired work functioning (due to health problems) is operationalized differently. Some, like the Work Productivity and Activity Impairment Questionnaire (WPAI), quantify it as hours or days being present at work, but with impaired functioning [20]. Others focus on the work roles, like the multidimensional Work Limitation Questionnaire (WLQ) [21], which differentiates the various aspects of work, i.e., time management demands, physical demands, mental and interpersonal demands, and output demands. What most instruments have in common is that they are designed to be generic, which allows them to be used in various different work settings. One newly developed instrument that distinguishes itself from the existing scales is the Nurses Work Functioning Questionnaire (NWFQ), which we have developed for nurses and allied health professionals [22]. Because of its job-specific nature, it better connects to the work context of nurses and allied health professionals. Its seven subscales capture domains of work that are relevant for these occupations. The job-specificity allows for the items to explicitly describe the concrete experiences and tasks of the work of nurses and allied health professionals. This characteristic of the items facilitates reflection on situations at work and enables self-report. It should be noted that NWFQ scores do not include an overall score of work functioning. Rather, the NWFQ gives insight into various aspects of the work of nurses and allied health professionals that might be impaired due to mental health complaints. The focus on work impairments related to mental health complaints is chosen as mental health complaints are expected to have, at least partly, different effects on work functioning than other (physical) health complaints, e.g., musculoskeletal disorders. For example, we know that mental health complaints can cause cognitive impairments, as the inability to concentrate can be one of the symptoms of impaired mental health. Therefore, we assume that different types of health complaints ask for different impaired work functioning questionnaires. Unlike other existing instruments measuring health-related work functioning, the items of the NWFQ do not explicitly refer to (known) health problems like the Work Limitation Questionnaire (WLQ) and the Stanford Presenteeism Scale (SPS) do [21], [23]. Three features of the NWFQ contribute to the usefulness on detecting of individuals with work functioning problems due to CMDs and of identifying the specific aspects of impaired work; thus, allowing for purposeful interventions. First, the job-specificity of the NWFQ items, second, the fact that these items do not refer to known health complaints and third the distinction of seven specific aspects of the work make detection of new cases by the NWFQ possible. Interventions that may be initiated based on the NWFQ scores might directly target the work e.g. temporary reorganization of work or discouraging the exertion of specific tasks, but might also address the employee's functioning and mental health complaints through guidance, support, or medical treatment by a (occupational) health professionals.

In an earlier study, some psychometric properties of the NWFQ were already evaluated i.e. the content validity, factorial validity and the internal consistency [22]. It has been shown that the NWFQ has high content validity, its subscales and items were evaluated as being comprehensive and relevant, and all subscales had acceptable to good internal consistency. Furthermore, its structural validity was good, as the subscale distribution was validated in a confirmatory factor analysis. However, other psychometric properties need to be evaluated further.

Regarding the reproducibility, which is the ability of a measurement tool to reproduce similar results in repeated measures of (stable) subjects, two aspects were evaluated in this study: the level of agreement and the test-retest reliability [24]. The level of agreement gives insight into the stability of the repeated scores within subjects. The test-retest reliability gives an indication of how well subjects can be distinguished from each other despite measurement errors.

In the present study, we also evaluated three types of construct validity [25]. First, we assessed convergent validity, which refers to the relationship between the tested instrument and instruments that measure related constructs. We chose to assess the relationship of the NWFQ with a generic work functioning questionnaire, as we expect them to overlap given that they both assess functioning at work. As the underlying construct of the NWFQ is “impaired work functioning due to mental health complaints”, we expect the NWFQ scores to be related to mental health problems. Therefore, we also assessed the relationship between the NWFQ-scores and the mental health complaints for the convergent validity. Second, we evaluated divergent validity to test for the non-relatedness with a measure of a dissimilar construct. For this purpose, we examined the association of the NWFQ subscales with a physical functioning scale, assuming that impairments in work functioning measured by the NWFQ are not related to pure physical health problems. Third, the discriminative validity was studied. As mental health problems are a probable cause of impairments in the work functioning, we expected to see differences between the groups of workers with and without mental health complaints.

In sum, the aim of this study was to assess the reproducibility of the NWFQ as well as its construct validity, encompassing convergent, divergent and discriminative validity.

## Methods

### Design

This study holds a within subject design with two measurement points: T1 and T2. The data from the first sample at T1 were used for the assessment of the convergent and discriminative validity. The data from the second sample at T2 were used for the divergent validity analysis, and the T2 data combined with the T1 data were used for the reproducibility analyses. The time interval between T1 and T2 was ten to 17 days, as during this span of time workers were expected to be stable with regard to work functioning and mental health.

### Subjects

A random sample of 1,200 nurses and allied health professions were contacted in one Dutch academic medical center in order to recruit 300 respondents. The expected response rate was low (25%) because of the large number of items in the questionnaire. The sample was stratified by occupation, gender, and age, and it was representative of the source population, which comprised all employed nurses (including surgical nurses and anesthetic nurses) and allied health professionals of that medical center. The sample at T2 consisted of the 300 employees who first completed the questionnaire at T1. We aimed to recruit 100 respondents for T2.

### Procedure

Data collection took place in August and September 2009. Prior to the distribution of the self-administered online questionnaire, the team managers of the relevant departments received information regarding the purpose, aim and procedure of the study. One week in advance of the distribution, all 1,200 eligible subjects were provided with general information about the study and its purpose through email. Two reminders were sent by email. The first 300 respondents of the questionnaire at T1 were emailed with a request to take part in the retest two weeks after they completed the questionnaire. After one week, a reminder was sent to these 300 subjects. Subjects were provided with an individual username and password to log in at the website with the questionnaire. Agreeing with the informed consent, which was shown online prior to the questionnaire, was a prerequisite for starting the questionnaire. Thus, all participants gave informed consent to participate in the described study.

It was possible to log out halfway through the survey and continue after logging in again. However, the questionnaire had to be fully completed within three days. It was not possible to skip questions. For each filled out questionnaire, we donated 2.50 Euro to a charity that the respondents could select from among three options.

The Medical Ethics Board of the Academic Medical Center Amsterdam gave exemption for ethical approval for the study.

### Instruments

#### Nurses Work Functioning Questionnaire (NWFQ)

The questionnaire tested in this study is the NWFQ developed by Gärtner and colleagues [22]. The NWFQ aims to measure impaired work functioning due to CMDs in nurses and allied health professionals. This 50-item self-report questionnaire consists of seven subscales: 1) *cognitive aspects of task execution and general incidents*, 2) *impaired decision making*, 3) *causing incidents at work* (not suitable for allied health professionals), 4) *avoidance behavior*, 5) *conflicts and annoyances with colleagues*, 6) *impaired contact with patients and their family*, and 7) *lack of energy and motivation*. Cronbach's alphas vary between 0.70 and 0.94. For the alpha values per scale, see results section. All items of the NWFQ have a reference period of four weeks. Response formats vary between 5-category and 7-category scales; however, the number of categories is the same for all items of one subscale. The content of the response scales varies between Likert-type scales (0 = *totally disagree* to 6 = *totally agree*; 0 = *disagree* to 4 = *agree*; 0 = *no difficulty* to 6 = *great difficulty*), relative frequency categories (0 = *almost never* to 6 = *almost always*; *0 = almost never* to 4 = *almost always*), and absolute frequency categories (0 = *not once* to 6 = *in general more than once a day*). In the present study, in addition to the specific response format for each item, a response category of ‘*Does not apply to my job*’ was also provided. In the calculation, this answer was treated as a missing value. The sum scores of the subscales ranged from 0–100 and were calculated as follows: (sum of item scores * 100) / (number of items of the subscale * maximum item score). For a more complete description of the development of the questionnaire, the content validity and the factorial structure, see Gärtner et al. [22].

#### Endicott Work Productivity Scale (EWPS)

This general work functioning scale is a 25-item self-report questionnaire with a five-point response scale (1 = *never*, 5 = *almost always*). The sum score is calculated following the traditional scoring method (0, 1, 2, 3, 4), ranging from 0 (best possible score) to 100 (worst possible score). The EWPS is valid and reliable, with a test-retest reliability (10 days to 2 weeks) of ICC = 0.92 and an internal consistency of α = 0.92 [26].

#### General Health Questionnaire (GHQ-12)

This self-report questionnaire was developed to detect common mental disorders in the general population [27]. Its 12 items have a four-point response scale corresponding to the symptoms present (1 = *not at all*, to 4 = *much more than usual*). The reference period for these items was “*the past days*”. For the sum score calculation, the traditional GHQ scoring method was used (0, 0, 1, 1), with a range of 0–12. Following earlier studies in working populations, a cut-off point of ≥4 was applied to identify individuals reporting sufficient psychological distress to be classified as probable cases of minor psychiatric disorder [28].

#### Four-Dimensional Symptoms Questionnaire (4DSQ)

Of the 4DSQ, which was developed to assess common mental health complaints, the 16-item distress subscale was used [29], [30]. The 4DSQ had a reference period of “*the past week*” and a five-point response scale (0 = *no*, 4 = *very often*). The internal consistency of the distress subscale in a working population was α = 0.90 [31]. For the sum score calculation, we followed the traditional scoring method (0, 1, 2, 2, 2) to generate a continuous distribution ranging from 0–32, where high scores indicated higher stress complaints. For case identification, a cut-off point of ≥11 was applied [32].

#### SF-36 physical functioning subscale

This physical functioning self-report scale contained ten items with a three-point response format (1 = *Yes, limited a lot*, 2 = *Yes, limited a little*, 3 = *No, not limited at all*) [33], [34]. A sum score was calculated to generate a continuous distribution ranging from 0 (worst health status) to 100 (best health status), using the formula (((sum of raw scores−10)/20) * 100). The SF-36 is valid and reliable; for the physical functioning scale, the internal consistency is α = 0.93, and the test-retest reliability (2 weeks) is r = 0.81 [35].

Demographic data were obtained for each employee. We assessed gender, age, family situation, ethnical background, occupation, number of work hours, labor contract, and years of work experience.

### Psychometric analyses

#### Test-retest reproducibility

For the test-retest reproducibility, we analysed the Level of agreement and the Test-retest reliability. For the level of agreement we assessed the absolute measurement errors. Therefore, we calculated the standard error of measurement (SEM) for each subscale [24]. The SEM equals the square root of the error variance of an ANOVA analysis, including systematic differences: SEM = √ (σ^2^_time_+σ^2^_error_). The SEM values indicate that if a within-subject comparison is made for two sum scores on a subscale of the NWFQ at different points in time, and a change score is smaller than the SEM, then it should be considered a measurement error. To visualize the level of agreement, a Bland and Altman plot with 95% confidence interval was designed [36], [37]. These plots show the difference scores of the subject in relation to the mean score of the test and retest. In the plots the mean change score and the 95% limits or agreement are illustrated. To detect possible systematic errors, the Pearson's correlation coefficients of the difference scores and the mean scores are given, as well a t-test is performed, to see if the mean change score significantly differs from zero [38].

The test-retest reliability evaluates the ability of the NWFQ to distinguish between subjects despite measurement error. Therefore, the intraclass correlations coefficient (ICC) using the T1 and T2 data (N = 212) was computed for all subscales. To determine the ICC a two-way random effects model was used, the ICC(A.1) according to MCGraw and Wong [39]. The ICC calculation method in which systematic differences are considered to be part of the measurement error was used, called the ICC absolute agreement. The formula used was: ICC = σ^2^_p_/(σ^2^_p_+σ^2^_time_+σ^2^_error_) [24]. For the ICC, we expected a minimum of 0.70 as sufficient for good reliability [37].

An assumption in reproducibility analyses is that the sample used is stable regarding the studied concept [37]. We expected our sample to be stable during the two weeks interval. To control for stability, we asked the participants: “*Did your state of well-being change after you first filled out our questionnaire?*” at the second measurement point. Subjects who answered “*no*” were regarded as stable subjects. The level of agreement and reliability analyses were performed separately for the whole sample and the sample with the stable subjects only. However, conclusions were based on the results of the stable sample only.

#### Construct validity

Threes types of construct validity were assessed, convergent validity, divergent validity and discriminative validity. The convergent validity was assessed by calculating correlations between the NWFQ subscales and the EWPS. As the NWFQ data were not normally distributed, Spearman correlations were used. For good convergent validity, we expected moderate (>0.30≤0.60) to high (>0.60) correlations in a positive direction for the relationship with the EWPS [40].

The divergent validity was assessed by calculating Spearman correlations between the NWFQ and the SF36 physical functioning scale. We chose the physical functioning subscale because its construct is dissimilar to the construct of the NWFQ, though it is not completely unrelated as both constructs refer to functioning of subjects. For good divergent validity, we expected these correlations to be low (≤0.30) [40].

Discriminative validity was assessed to evaluate the ability of the NWFQ subscales to discriminate between groups that were expected to differ. Therefore, we used a Mann Whitney U test to test for significant differences in NWFQ scores between workers with and without mental health complaints. Having mental health complaints was defined as scoring above the cut-off on one or both of the mental health complaints scales (GHQ-12 and 4DSQ-distress). To correct for the high number of tests performed (one for each of the seven subscales), we used a Bonferroni adjustment. Therefore, p<0.007 was regarded as significant.

## Results

Of the 1,200 nurses and allied health professionals invited, 314 employees fully completed the questionnaire at the first measurement point (26% response rate). Of these 314, 112 (36%) completely filled out the second questionnaire (T2). Table 1 presents the socio-demographic characteristics of the samples at T1 and T2.

**Table 1:** Socio-demographic characteristics of the two samples.

Demographic characteristics	T1 (N = 314)	T2 (N = 112)
*Gender (N (%))*		
Female	257 (81.8)	94 (83.9)
Male	57 (18.2)	18 (16.1)
Age in years (mean (SD))	44.5 (12.0)	46.3 (10.5)
*Marital status (N (%))*		
Married/ living together with a partner	227 (72.3)	84 (75)
In a relationship	21 (6.7)	8 (7.1)
Single	54 (17.2)	15 (13.4)
Divorced	11 (3.5)	5 (4.5)
Widow/ widower	1 (0.3)	0 (0)
*Ethnical background (N (%))*		
Dutch	261 (83.1)	94 (83.9)
Immigrant first generation	35 (11.1)	13 (11.6)
Immigrant second generation	18 (5.7)	5 (4.5)
*Occupation (N (%))*		
Nurse	220 (70.1)	74 (66.1)
Surgical nurse	23 (7.3)	7 (6.2)
Anesthetic nurse	13 (4.1)	6 (5.4)
Allied health professional	58 (18.5)	25 (22.3)
Working experience in years (mean (SD))	20.8 (12.2)	22.3 (11.6)
*Labor contract (N (%))*		
Permanent position	301 (95.9)	107 (95.5)
Fixed-term contract	9 (2.9)	1 (0.9)
Temporary employment	4 (1.3)	4 (3.6)
Work hours per week (mean (SD))	30 (6.3)	29 (6.8)

### Reproducibility

Twenty-eight subjects responded that their well-being improved or deteriorated between the two measurement points. Therefore, we did not regard them as stable enough to include in our reproducibility analyses.

#### Level of agreement

**Figure 1:**
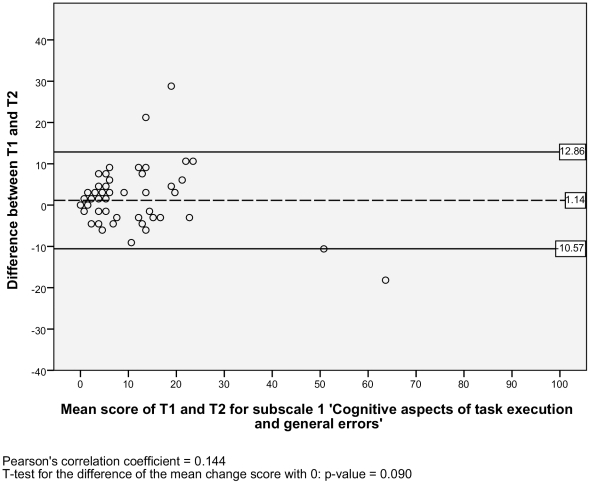
Bland and Altman plot for subscale 1 ‘Cognitive aspects of task execution and general incidents’.

**Figure 2:**
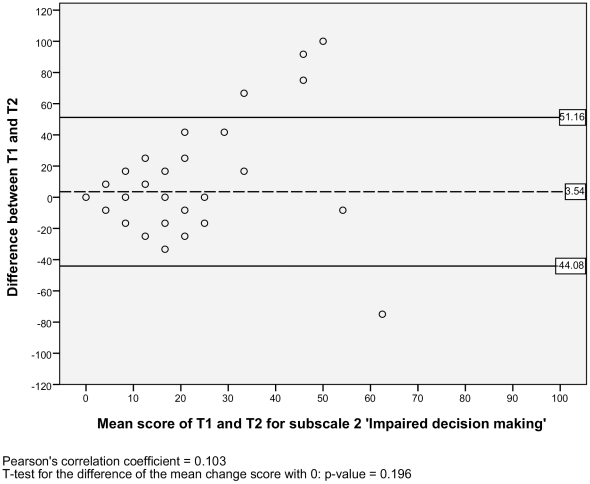
Bland and Altman plot for subscale 2 ‘Impaired decision making’.

**Figure 3:**
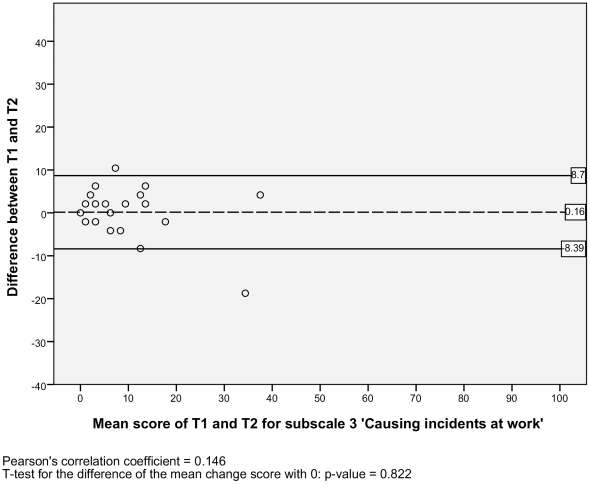
Bland and Altman plot for subscale 3 ‘Causing incidents at work’.

**Figure 4:**
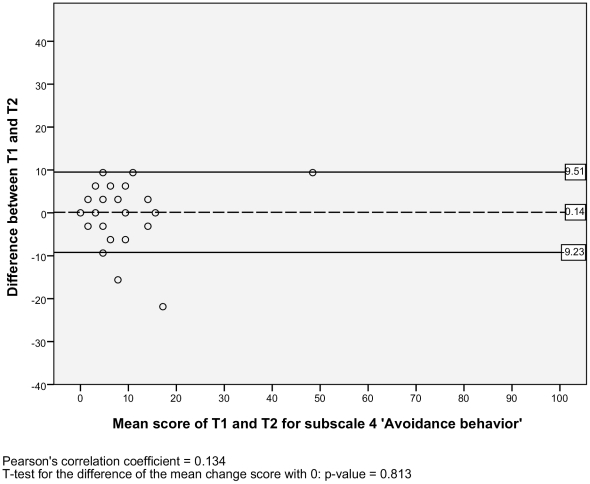
Bland and Altman plot for subscale 4 ‘Avoidance behavior’.

**Figure 5:**
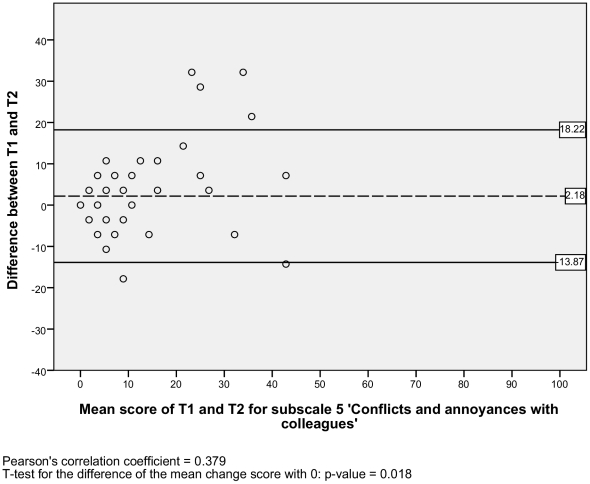
Bland and Altman plot for subscale 5 ‘Conflicts and irritations with colleagues’.

**Figure 6:**
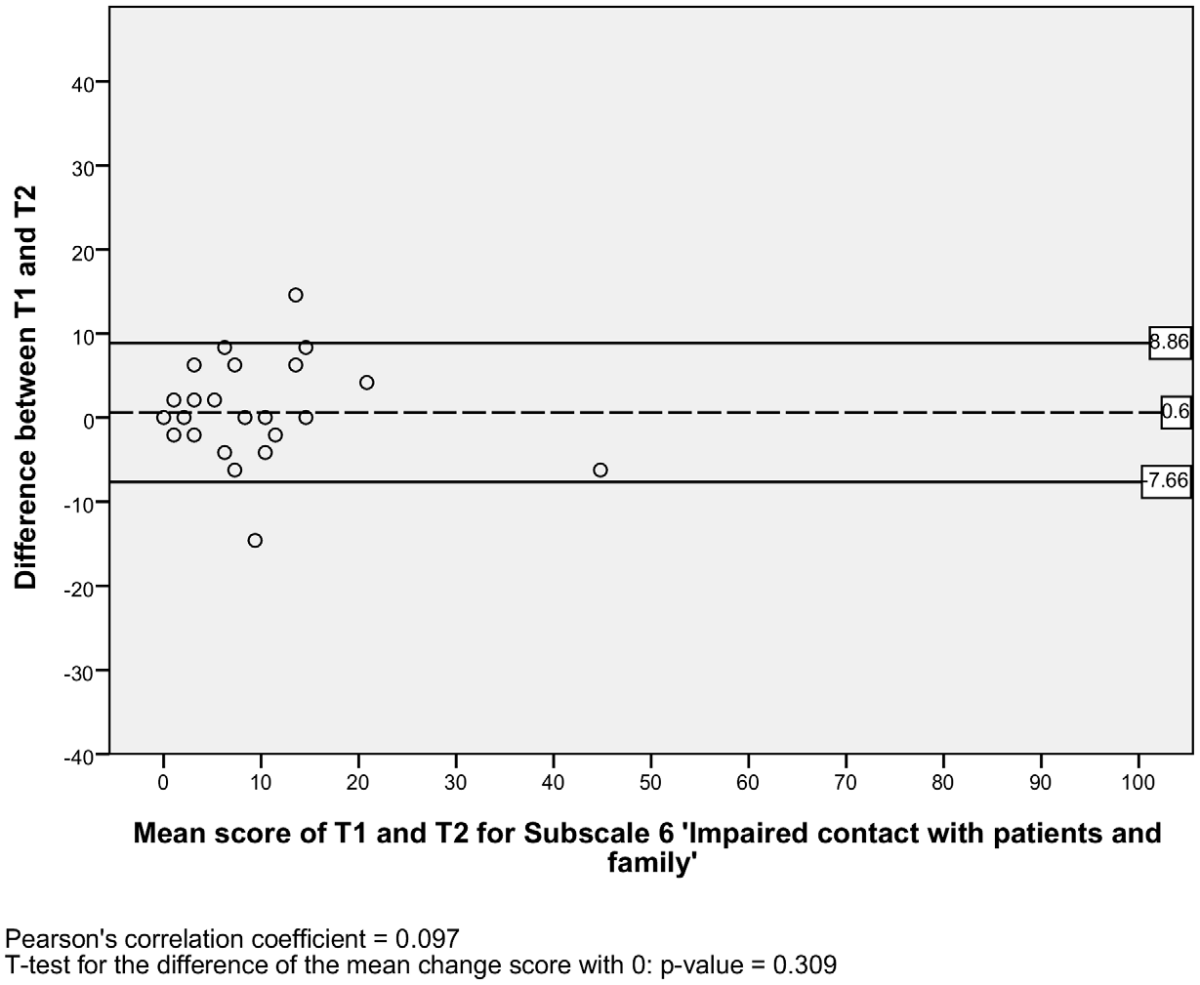
Bland and Altman plot for subscale 6 ‘Impaired contact with patients and their family’.

**Figure 7:**
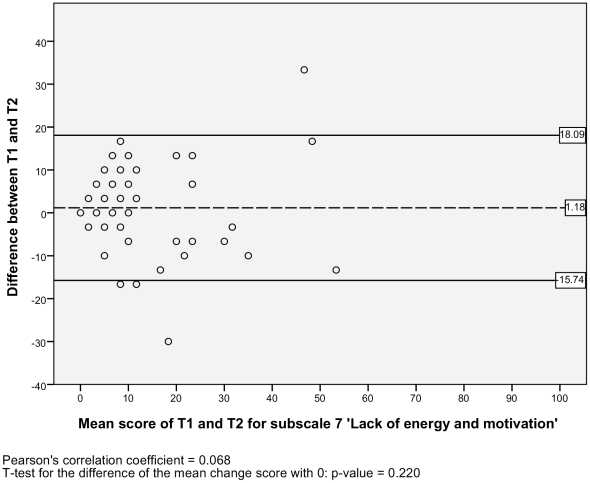
Bland and Altman plot for subscale 7 ‘Lack of energy and motivation’.

The SEM ranged from 2.95 to 6.12 for the six subscales, and there was one outlier with a SEM of 17.11 for the subscale 2) *impaired decision making* (Table 2). Additionally, the Bland and Altman plots are shown for each NWFQ subscale, based on the stable sample (Figure 1, 2, 3, 4, 5, 6, 7). The Bland and Altman plots show the difference in NWFQ scores at the two measurement points. The dots in the figure present the difference scores of the subjects and the lines picture the 95% confidence interval. The 95% confidence intervals vary between the subscales, for four subscales they range from about −8 to 13, for two subscales they are a little larger about −14 to 18. For subscale 2) *impaired decision making*, the 95% confidence interval is very large, with −44 to 51. In all subscales except for subscale 5) *conflicts and annoyances with colleagues*, the mean change score is close to zero and no significant correlation between the mean scores of T1 and T2 and the difference scores are found. In subscale 5) *conflicts and annoyances with colleagues*, the mean difference score of 2.18 statistically differs from zero with a p-value of 0.018, the correlation coefficient of the mean scores of T1 and T2 and the difference scores is 0.379.

**Table 2:** ICC and SE of the sample who completed both questionnaires at T1 and T2.

NWFQ subscales	First measure (T1)			Second measure (T2)							ICC 95% CI	
	Valid N	Median (range)		Valid N	Median (range)		Mean difference of T1–T2 (SD)		SEM	ICC	Lower bound	Upper bound
**Stable sample (N = 84)**												
1. Cognitive aspects of task execution and general incidents	81	3	(0–55)	83	2	(0–73)	1.14	(5.98)	4.27	**0.85**	0.77	0.90
2. Impaired decision making	81	0	(0–100)	83	0	(0–100)	3.54	(24.30)	17.11	0.16	−0.05	0.37
3. Causing incidents at work	41	2	(0–40)	41	0	(0–44)	0.16	(4.36)	3.04	**0.88**	0.79	0.94
4. Avoidance behavior	75	0	(0–53)	74	3	(0–44)	0.14	(4.78)	3.33	**0.79**	0.69	0.87
5. Conflicts and annoyances with colleagues	83	4	(0–50)	83	9	(0–50)	2.18	(8.19)	6.03	**0.72**	0.59	0.81
6. Impaired contact with patients and family	61	2	(0–42)	61	2	(0–48)	0.60	(4.21)	2.95	**0.86**	0.76	0.91
7. Lack of energy and motivation	82	7	(0–63)	84	3	(0–60)	1.18	(8.63)	6.12	**0.74**	0.63	0.83
**Total sample (N = 112)**												
1. Cognitive aspects of task execution and general errors	107	5	(0–55)	111	3	(0–73)	0.20	(8.78)	6.17	**0.70**	0.59	0.79
2. Impaired decision making	109	0	(0–100)	111	0	(0–100)	3.86	(21.90)	15.58	0.32	0.14	0.48
3. Causing incidents at work	58	2	(0–40)	58	2	(0–44)	0.15	(5.04)	3.52	**0.82**	0.71	0.89
4. Avoidance behavior	100	0	(0–53)	100	0	(0–44)	0.27	(6.33)	4.41	0.66	0.52	0.76
5. Conflicts and annoyances with colleagues	111	4	(0–50)	111	4	(0–61)	1.14	(9.50)	6.79	0.67	0.56	0.76
6. Impaired contact with patients and family	80	2	(0–42)	82	2	(0–48)	1.42	(7.54)	5.30	0.60	0.42	0.73
7. Lack of energy and motivation	109	7	(0–63)	111	3	(0–60)	0.76	(8.38)	5.92	**0.78**	0.70	0.85

Bold printed = values support the hypotheses.

In the plot of subscale 2) i*mpaired decision making*, several high change scores can be seen, one is up to 100% of the scale with a change score of 100. Also for this subscale the confidence interval is high, its values are nearly half of the scale range with 51.16 and −44.08.

#### Test-retest reliability

Based on the sample with stable subjects, the single measure ICC between measurements at T1 and T2 were good for six of the seven subscales, with a range of 0.72 to 0.88 (Table 2). Subscale 2) *impaired decision making* had a poor reliability score with an ICC of 0.16. Figure 1, 2, 3, 4, 5, 6, 7 present the Bland & Altman plots for each subscale.

### Construct validity

#### Convergent validity

The Spearman's rank correlation coefficients between the NWFQ subscales and the EWPS sum scale ranged from 0.22 to 0.62 (Table 3). There was one low correlation for subscale 2 (r = 0.22), five medium correlations for subscales 3, 4, 5, 6, and 7 and one high correlation for subscale 1 (r = 0.62).

**Table 3:** Overview of subscale characteristics and correlations for the construct validity analyses.

		T1 (total N = 314)					T2 (total N = 112)		
						Spearman's correlation			Spearman's correlation
NWFQ subscales	# of items	N	Cronbach's α	Median (range)		EWPS	N	Cronbach's α	SF36 physical functioning
1. Cognitive aspects of task execution and general incidents	11	308	.94	5	(0–82)	**0.62**	113	.94	**−0.19**
2. Impaired decision making	3	310	.88	0	(0–100)	0.22	113	.80	**−0.11**
3. Causing incidents at work	8	178	.78	4	(0–40)	**0.42**	60	.88	**−0.06**
4. Avoidance behavior	8	294	.70	0	(0–81)	**0.38**	102	.61	**−0.23**
5. Conflicts and annoyances with colleagues	7	311	.77	4	(0–61)	**0.49**	113	.74	**−0.11**
6. Impaired contact with patients and family	8	223	.81	4	(0–42)	**0.50**	83	.81	**−0.10**
7. Lack of energy and motivation	5	307	.81	7	(0–73)	**0.53**	113	.81	**−0.13**

Bold printed = correlations support the hypotheses.

#### Divergent validity

The Spearman's rank correlation coefficients between the subscales of the NWFQ and the SF-36 physical functioning sum score were all low, ranging from −0.23 to −0.06 (Table 3).

#### Discriminative Validity

Significant differences in the expected direction were found between the group with and without mental health complaints for all seven subscales (Table 4). The p-values ranged from p<0.001 to p = 0.003.

**Table 4:** Discriminative validity using a Mann-Whitney-U test with the T1 sample.

	Total			Healthy			CMD*			p-value of the Mann-Whitney-U test
NWFQ subscales	N	Median (range)		N	Median (range)		N	Median (range)		CMD*
1. Cognitive aspects of task execution and general incidents	308	5	(0–82)	227	3	(0–39)	81	11	(0–82)	**<0.001**
2. Impaired decision making	310	0	(0–100)	227	0	(0–100)	83	8	(0–92)	**0.003**
3. Causing incidents at work	178	4	(0–40)	130	2	(0–19)	48	6	(0–40)	**0.003**
4. Avoidance behavior	294	0	(0–81)	214	0	(0–31)	80	6	(0–81)	**<0.001**
5. Conflicts and annoyances with colleagues	311	4	(0–61)	228	0	(0–50)	83	14	(0–61)	**<0.001**
6. Impaired contact with patients and family	223	4	(0–42)	167	2	(0–29)	56	6	(0–42)	**0.001**
7. Lack of energy and motivation	307	7	(0–73 )	226	3	(0–63)	81	20	(0–73)	**<0.001**

Bold printed = significant difference between the two groups, above versus below the cut-off score using a Bonferroni-adjusted p-value of p<0.007.

*above cut-off for GHQ-12 and/or 4DSQ-distress.

## Discussion

The purpose of this study was to evaluate the psychometric quality of the newly developed NWFQ in terms of reliability and construct validity. Overall, the results were satisfactory for six of the seven subscales.

Except for subscale 2) *impaired decision making*, the subscales of the NWFQ had good reproducibility and thus were able to distinguish between subjects, even when measurement error was taken into account. The SEM values, expressed in the same value as the target instrument, help to interpret the changes in scores of individuals over time on the NWFQ subscales. When within subjects comparisons were made, changes had to be larger than the SEM to ensure that the observed differences were not due to measurement error. Based on the Bland and Altman plots, we can state that level of agreement is good for six of the seven subscales. For subscale 5) *conflicts and annoyances with colleagues* systematic error appears to influences the score. Based on the dots in the plot, we suppose a possible reason for systematic error might be that subjects with high mean scores at T1 tend to improve at T2, rather than being stable or deteriorate. In future studies on the characteristics of the NWFQ, such as assessment of the responsiveness, these possible systematic differences should be taken into account, e.g., by subgroup analyses in which analyses are stratified for groups of subjects with high and low baseline scores.

Our data offer strong support for good construct validity as the hypothesized relationships were confirmed, with the exception of subscale 2) *impaired decision making*. Regarding the convergent validity of these six subscales, all correlations with the EWPS were substantial and in line with the hypothesis. The fact that the correlations were medium and not high verifies that the NWFQ, on the one hand, has enough overlap with a generic work productivity scale. On the other hand, this job-specific instrument measures aspects of additional value compared to a generic questionnaire. Regarding divergent validity, the hypothesis that the correlations between the NWFQ and the unrelated physical functioning measure are low is supported for all the subscales. All scales showed clear discriminative validity; thus, they discriminate well between a group of subjects with and without mental health complaints. Therefore, the relatedness of CMDs with impaired work functioning is evident.

It is obvious that subscale 2) *impaired decision making* performed the weakest in our evaluation of the psychometric properties of the NWFQ. The subscale failed to show good reliability. In a subscale with only three items, small differences in scores on one item have bigger impact for the stability of the measures than in scales with more items. High reliability scores are therefore more difficult to derive in subscales with smaller number of items. However, increasing the number of items is no attractive alternative, as in the development process of the NWFQ, the three item option for this subscale led to the best internal consistency and interpretability. We therefore must conclude that in our sample, the subscale was not able to distinguish between individuals. In addition, the subscale *impaired decision making*, failed to support the hypothesis for good convergent validity. Consequently, we have to discourage the use of subscale 2) *impaired decision making* in the present form. However, we still regard impaired decision making to be an important aspect of the construct of the NWFQ. During the development process of the NWFQ, the aspect impaired decision making as an effect of CMDs was discussed repeatedly in focus groups with nurses and professionals. Additionally, in expert checks, the content validity of the subscale was confirmed and impaired decision making was evaluated as an important aspect of the overall construct.

### Methodological notes

A methodological limitation of this study that deserves consideration is that the data were primarily collected within the scope of the questionnaire development. The items of the tested subscales were included in the original item pool, which was much longer than the final version of the NWFQ. Therefore, the context of questionnaire administration was not exactly the same as it will be in future use. This is evident in the low response rate for our study, which can partly be explained by the length of the questionnaire administered. However, the sample was representative for the gender and age distribution of the studied occupations in the medical center.

### Recommendations for further research

We want to point out three aspects for further research. The first point concerns the subscale 2) *impaired decision making*. As described above, we regard that subscale as necessary part of the NWFQ; however, no reproducible and valid form of measuring that subsconstruct is found yet. Therefore, future research should identify new items measuring impaired decision making in nurses and allied health professionals with CMDs that form a subscale with good psychometric quality. Second, the conclusion drawn from the presented data is only valid for the Dutch version of the NWFQ and for nurses and allied health professionals working in academic medical centers. Although a backward-forward translation of the questionnaire into English exists, we recommend additional evaluations of the psychometric quality of that version. Third, future use of the NWFQ as a diagnostic instrument in occupational health practice, suitable cut-off values for the subscales need to be identified. In addition, the responsiveness, the smallest detectable change (SDC) and minimal important change (MIC), would be important to assess as they would allow for making inferences based on the changes in scores of individual workers on the NWFQ over time.

### Recommendations for practice

Until now, work functioning instruments have mainly been used in the scientific setting for research aims. It would be of great value to apply them for use in occupational health practice as well, and in particular, applying the NWFQ for preventive aims would be of value. For preventive purposes, work functioning instruments must help to realize two aims; first, timely interventions and second, purposeful interventions on mental health complaints and related impairments in the functioning. We conclude that the nature of the NWFQ fulfills both these needs. The NWFQ can be used for detection purposes as its items do not refer to known health problems; furthermore, its multidimensionality makes identification of specific work aspects possible, and therefore is a starting point for purposeful interventions. One possible way to initiate the detection and monitoring of employees with mental health complaints and related work functioning problems for preventive purposes would be in a Workers' Health Surveillance in which the NWFQ could be included.

The NWFQ is available for use, see supporting file S1. Though, users have to follow Creative Commons Attribution-ShareAlike 3.0 Unported (CC BY-SA 3.0).

### Conclusion

The NWFQ demonstrated good psychometric properties for six subscales. Subscale 2) *impaired decision making*, did not show enough ability to discriminate between subject and the association with other work functioning measure was too weak; therefore, we discourage use of that subscale in the present form. In conclusion, the NWFQ is a reproducible and valid instrument suitable for the measurement of impairments in work functioning due to CMDs in nurses and allied health professionals when including six of the seven subscales.

## Supporting Information

File S1 **The Nurses Work Functioning Questionnaire.**
(PDF)
