# Supplementary material for: Psychometric Properties of the Nurses Work Functioning Questionnaire (NWFQ)
Source: PLoS One. 2011 Nov 8;6(11):e26565. doi: 10.1371/journal.pone.0026565 (PMC3210752; doi:10.1371/journal.pone.0026565)
Supplement: File S1 — The Nurses Work Functioning Questionnaire. (PDF) [file pone.0026565.s001.pdf]

Supporting file S1

The Nurses Work Functioning Questionnaire (NWFQ)

The Nurses Work Functioning Questionnaire

To what extent did you experience difficulty in meeting the following aspects **of your work** in the **past 4 weeks**?

		**no difficulty**						**great difficulty**
1	Keeping sufficient overview of your tasks.	□	□	□	□	□	□	□
2	Not forgetting something one day.	□	□	□	□	□	□	□
3	Working efficiently.	□	□	□	□	□	□	□
4	Carrying out your work activities in general.	□	□	□	□	□	□	□
5	Carrying out your work independently.	□	□	□	□	□	□	□
6	Staying alert in your work.	□	□	□	□	□	□	□
7	Working accurately.	□	□	□	□	□	□	□
8	Preventing incidents in your work.	□	□	□	□	□	□	□
9	Working carefully.	□	□	□	□	□	□	□
10	Contact with patients and their family.	□	□	□	□	□	□	□
11	Showing sufficient empathy towards patients and their family.	□	□	□	□	□	□	□
12	Taking time for your patients.	□	□	□	□	□	□	□
13	Competently handling aggressive patients or family members.	□	□	□	□	□	□	□
14	Error-free administering/handing medication.	□	□	□	□	□	□	□

Relate the following statement **to your work** in the **past 4 weeks**. How much do you agree with it?

		**totally disagree**	**disagree**	**disagree a little**	**not agree/ not disagree**	**agree a little**	**agree**	**totally agree**
15	I make mistakes more often than before.	□	□	□	□	□	□	□

With respect to the **past 4 weeks**, are you someone who…

		**(almost) never**	**once in a while**	**some-times**	**regularly**	**often**	**very often**	**(almost) always**
16	... has the feeling to have lost control over the work?	□	□	□	□	□	□	□
17	… does not look forward to a working day/ shift?	□	□	□	□	□	□	□
18	… starts the working day/ shift already moaning and groaning?	□	□	□	□	□	□	□
19	... works without any enthusiasm?	□	□	□	□	□	□	□

How often did you think the following in the **past 4 weeks**?

20	I have great difficulty in getting through a working day.	□	□	□	□	□	□	□
21	I have the need for an extra day off to be able to get through the working week well.	□	□	□	□	□	□	□

How often did the following situations occur **in your work** in the **past 4 weeks?**

22	I do not succeed in listening well to my patients.	□	□	□	□	□	□	□
23	I notice myself that I treat patients too roughly.	□	□	□	□	□	□	□

How often were the following situations **with respect to your work** applicable to you in the **past 4 weeks**?

		**not once**	**1 x per month**	**2 - 3 x per month**	**on average 1 x per week**	**on average 2 - 3 x per week**	**on average 1 x per day**	**on average more than 1 x per day**
24	The tone of voice I use with my patients or their family is not too friendly.	□	□	□	□	□	□	□
25	I am curt towards patients or their family.	□	□	□	□	□	□	□

Answer the questions below with respect to the **past 4 weeks.** **How often did you...**

26	… almost caused incidents in your work?	□	□	□	□	□	□	□
27	… almost made a mistake in the administration/handing of medicines?	□	□	□	□	□	□	□

28	… underestimated the seriousness of a situation?	□	□	□	□	□	□	□

How often did something go wrong while you were carrying out the tasks below in the **past 4 weeks?**

29	Initiating infusion.	□	□	□	□	□	□	□
30	Assessing which (nursing) care a patient needs.	□	□	□	□	□	□	□
31	Performing and reporting actions (e.g. in the nursing plan or treatment plan).	□	□	□	□	□	□	□

How often did you **almost** do something wrong while carrying out the tasks below in the **past 4 weeks**?

32	Performing and reporting actions (e.g. in the nursing plan or treatment plan).	□	□	□	□	□	□	□

Indicate how often in the **past 4 weeks** the following has happened.

		**(almost) never**	**some-times**	**regularly**	**often**	**(almost) always**
33	Friction between you and someone from your team.	□	□	□	□	□
34	Being irritated during work.	□	□	□	□	□
35	Tensions between you and your colleagues.	□	□	□	□	□

How often was the behavior below applicable to you in the **past 4 weeks**?

36	Leaving for a moment so you can be alone.	□	□	□	□	□
37	Avoiding conversations with your colleagues.	□	□	□	□	□
38	Avoiding common areas.	□	□	□	□	□
39	Avoiding working together with your colleagues.	□	□	□	□	□

Relate the following statements **to your work** in the **past 4 weeks**. How much do you agree with it?

		**disagree**	**disagree a little**	**not agree/ not disagree**	**agree a little**	**agree**
40	Making decisions I leave to my colleagues.	□	□	□	□	□
41	I often only do what is absolutely necessary.	□	□	□	□	□
42	Meetings and evaluations I avoid as much as possible.	□	□	□	□	□
43	I prefer to do only routine jobs.	□	□	□	□	□
44	I am often astonished at how easy it is for others to upset me.	□	□	□	□	□
45	I often react irritated towards colleagues/ managers.	□	□	□	□	□
46	I often get angry about matters at work.	□	□	□	□	□
47	I often have conflicts with my manager.	□	□	□	□	□
48	I was able to make important decisions in my work responsibly. (R)	□	□	□	□	□
49	I can quickly decide how to handle matters in my work. (R)	□	□	□	□	□
50	I know how to make the right decisions in stressful situations. (R)	□	□	□	□	□

Table 1

Instructions for sum score calculation

	Subscales	Items	Calculation of standardized sum score	# of items total	# of items minimum
1	Cognitive aspects of task execution and general incidents	1, 2, 3, 4, 5, 6, 7, 8, 9, 15, 16	(sum of item scores * 100) / (# of items * 6)	11	9
2	Impaired decision making*	48(R), 49(R), 50(R)	(sum of item scores * 100) / (# of items * 4)	3	3
3	Causing incidents at work**	14, 26, 27, 28, 29, 30, 31, 32	(sum of item scores * 100) / (# of items * 6)	8	6
4	Avoidance behavior	36, 37, 38, 39, 40, 41, 42, 43	(sum of item scores * 100) / (# of items * 4)	8	6
5	Conflicts and irritations with colleagues	33, 34, 35, 44, 45, 46, 47	(sum of item scores * 100) / (# of items * 4)	7	6
6	Impaired contact with patients and their family	10, 11, 12, 13, 22, 23, 24, 25	(sum of item scores * 100) / (# of items * 6)	8	6
7	Lack of energy and motivation	17, 18, 19, 20, 21	(sum of item scores * 100) / (# of items * 6)	5	4

Technical details:

- Items followed by (R) need to be recoded before sum score is calculated.
- Item score counting starts with 0 on the outer left category, add 1 point for each category further to the right (e.g. *disagree* = 0; *disagree a little* = 1; *not agree/ not disagree* = 2; *agree a little* = 3; *agree* = 4).
- Calculation of standardized sum scores follows the principle: (sum of item scores * 100) / (# of items * maximum score per item).
- For sum scores calculation, subjects need to have filled out at least ¾ of all items of a subscale.
- The range of the standardized sum score is 0-100 for each subscale.

* We discourage use of the subscale impaired decision making in the present form, based on reproducibility and validity analyses.

**The subscale “Causing incidents at work” is not suitable for allied health professionals.

Every body is free to use the NWFQ taking into account
Creative Commons Attribution-ShareAlike 3.0 Unported (CC BY-SA 3.0):

You are free:
to Share — to copy, distribute and transmit the work
to Remix — to adapt the work to make commercial use of the work
Under the following conditions:
Attribution — You must attribute the work in the manner specified by the author or licensor (but not in any way that suggests that they endorse you or your use of the work).
Share Alike — If you alter, transform, or build upon this work, you may distribute the resulting work only under the same or similar license to this one.

For the full description of the legal code see: http://creativecommons.org/licenses/by-sa/3.0/
